# Requiring durations of therapy at the time of antibiotic order entry reduces antibiotic use

**DOI:** 10.1017/ash.2025.20

**Published:** 2025-02-12

**Authors:** Megan R. Wright, Jessica Gillon, Sophie E. Katz, Ritu Banerjee

**Affiliations:** 1 Department of Pharmaceutical Services, Vanderbilt University Medical Center, Nashville, TN, USA; 2 Division of Pediatric Infectious Diseases, Vanderbilt University Medical Center, Nashville TN, USA

## Abstract

The impact of required durations of therapy for antibiotic orders at the time of order entry has not been reported. Requiring ordering clinicians to enter stop dates at the time of antibiotic order entry decreased DOT/1000 patient days for orders with empiric indication from 154 to 119 (–34.9 (–55.7 to –14)).

## Introduction

Over 50% of antibiotics prescribed in the inpatient setting are not consistent with recommended prescribing guidelines, and often prescribed for excessively long durations.^1^ Requiring providers to enter a duration of therapy at the time of antibiotic ordering may impact antimicrobial utilization. In May 2022, we implemented an electronic medical record change that required providers to enter a duration of therapy, in doses, hours or days, at the time of antibiotic order entry for all pediatric inpatients. We hypothesized that this requirement would lead to shorter durations of therapy.

## Methods

This was a retrospective, pre- to post-evaluation of children (18 years or younger) who received antibiotics while admitted to an inpatient unit at a tertiary care free-standing academic children’s hospital. The pre-intervention period was June through November 2020 and 2021 and the post-intervention period was June through November 2022. Two pre-intervention periods were chosen to help mitigate changes in infection patterns related to the COVID-19 pandemic and to account for seasonal variability in antibiotic use given the limited months in the post-intervention period. We reviewed antibiotic medication orders with an indication of empiric treatment for suspected infection, respiratory tract infection, intrabdominal/gastrointestinal infection, or skin and soft tissue infection (selected as they represent approximately 60% of antibiotic use in our hospital). Patients were excluded if antibiotics were ordered without selecting an indication or were only ordered in the emergency department or operating room.

The primary outcome was normalized days of therapy (DOT) by indication, per 1000 days present (PD; National Healthcare Safety Network (NHSN))^2^. Secondary outcomes included DOT/1000 PD according to the NHSN classifications: community-onset antibiotics, hospital-onset antibiotics, resistant gram-positive antibiotics, and other.^2^ Vancomycin was removed from the resistant gram-positive classification and analyzed independently. In addition to requiring stop dates at the time of order entry, other antimicrobial stewardship (ASP) initiatives did occur during all three time periods. Statistical analysis with the independent *T*-test was completed using IBM SPSS Version 29. This study was approved by the Vanderbilt University Institutional Review Board.

## Results

During the study period 8524 patients received antibiotics. We excluded 3697 patients due to missing indication (372 patients in the pre-intervention period) or an indication outside the inclusion criteria (2564 and 1403 patients in the pre- and post-intervention period, respectively), resulting in a study sample of 4827 patients. The pre- and post-intervention groups were similar in terms of age at time of administration (1.08 pre vs. 2.05 post), but thirty-day readmission rate numerically decreased from 10% to 8% over the study period. There was a mean difference of -20.5 (95% CI, 12.7 to – 47.5) in overall DOT, though this was not statistically significant. DOT/1000 PD for empiric indications was significantly lower in the post-intervention period with a mean difference of –34.9 (95% CI, –55.7 to –14; (Table 1). There was a non-significant increase in DOT/1000 PD for intra-abdominal/gastrointestinal infections (36 vs 41 (Mean Difference 4.8 (95% CI, –5.3 to 14.8). For respiratory tract infection, an increase in DOT/1000 PD from 42 to 50 (mean difference 7.9 (95% CI, –6.1 to 21.9)) was observed. Further analysis revealed an increase in the number of patients with respiratory infection indications in the post-intervention group. The median duration of therapy for respiratory tract infections was 6 days (IQR 3–13) in 2020 and 4 days (2–8) in both 2021 and 2022.

**Table 1.** DOT per 1000 days present by indication and NHSN classification

	Pre (2020–2021) n = 3004 mean (SD)	Post (2022) n = 1803 mean (SD)	Mean difference (95% CI)
Indication			
Empiric treatment for suspected infection	154 (16)	119 (26)	–34.9 (–55.7 to –14)
Intra-abdominal/Gastrointestinal infection	36 (9)	41 (10)	4.8 (–5.3 to 14.8)
Respiratory tract infection	42 (10)	50 (18)	7.9 (–6.1 to 21.9)
Skin and soft tissue infection	20 (3)	19 (4)	–1.2 (–4.6 to 2.3)
Total for included indications	250 (25)	229 (27)	–20.5 (12.7 to –47.5)
NHSN Classification			
Community-onset antibiotics	72 (7)	75 (9)	2.9 (–5 to 10.9)
Hospital-onset antibiotics	79 (12)	64 (15)	–15.1 (–29 to –1.3)
Resistant gram-positive antibiotics^a^	15 (4)	17 (7)	2.4 (–2.6 to 7.4)
Vancomycin	33 (6)	28 (3)	–5.4 (–9.6 to –1.3)
Other	50 (8)	45 (9)	–5.4 (–14 to 3.3)

a Excludes vancomycin.

Among secondary outcomes, there was an overall decrease from pre-intervention to post-intervention periods in DOT/1000 PD of hospital-onset antibiotics (Mean Difference –15.1 (95% CI, –29 to –1.3) as well as vancomycin (Mean Difference –5.4 (95% CI, –9.6 to –1.3) (Table 1). Use of community-onset antibiotics was numerically higher in the post-intervention group (Mean Difference 2.9 (95% CI, –5 to 10.9)). DOT/1000 PD for resistant Gram-positive antibiotics excluding vancomycin was not different between the periods. Hospital demographics (case mix index, patient race/ethnicity, and insurance type) did not differ across the entire hospital during the study period.

## Discussion

To our knowledge, this is the first paper assessing the impact of required antibiotic durations at time of order entry on antibiotic DOT. After requiring documentation of discontinuation dates for inpatient antimicrobial orders, DOT/1000 PD was significantly for orders with an empiric indication. The large reduction in DOT/1000 PD for empiric treatment may be due to the intervention or due to an increase in selection of the appropriate indication. Unpublished institutional data from the same time period showed the rates of accurate indication selection increased over the study period. It is the authors’ opinion that clinicians were more thoughtful in indication selection and updating orders as treatment changed leading to fewer definitive treatment courses with an empiric therapy indication. A limitation of using DOT/1000 PD as a marker of durations of therapy is that an increase in the number of antibiotic utilized for a shorter duration will cause and increase in the DOT/1000 PD. For example, a patient receiving one day piperacillin/tazobactam is 1 DOT where cefepime and metronidazole would be 2 DOT for the same duration. This limitation could be the reason for the increases seen in both abdominal/gastrointestinal infections and respiratory tract infections. The increase in DOT/1000 PD for intra-abdominal/gastrointestinal infections may be attributed to a change in clinician prescribing practices. In the pre-intervention period, piperacillin-tazobactam was predominantly ordered for this indication, while during the post-intervention group, clinicians used combination therapy with cefepime and metronidazole more frequently, thereby increasing the DOT/1000 PD. The increase in DOT/1000 PD for respiratory tract infection could be due to the increased prevalence of dual coverage for respiratory tract infections in the post-intervention group. During the post-intervention period, it was more common for providers to utilize dual MRSA coverage with clindamycin and vancomycin than in the pre-intervention period. The decreased duration of therapy further suggests that the rise in DOT/1000 PD could be due to increased utilization of multiple antibiotics empirically.

This study has several limitations. It is a single center, retrospective descriptive study that may not be generalizable to other institutions. Concurrent ASP interventions (eg, implementation of MRSA nasal PCR to aid vancomycin de-escalation and dissemination of an updated local pneumonia guideline recommending shorter duration of therapy) may have impacted treatment for Gram positive respiratory infections. The study period encompassed the COVID-19 pandemic, which led to increased acuity of hospitalized patients, decreased in-person ASP rounds, and disrupted seasonal patterns of many infections like influenza and respiratory syncytial virus.^3^ We were also unable to determine the number of missed antibiotic doses and unintentional discontinuations. Despite these limitations, the ASP team performed almost daily audit and feedback and did not identify any harms from unintentional antibiotic discontinuation.

We demonstrate that requiring clinicians to enter stop dates at the time of antibiotic order entry, combined with an active ASP, was associated with decreased antibiotic utilization.
